# (*R*)-Di-*tert*-butyl 1,1′-binaphthyl-2,2′-dicarboxyl­ate

**DOI:** 10.1107/S1600536808044309

**Published:** 2009-01-08

**Authors:** Melanie Thoss, Rüdiger W. Seidel, Martin Feigel

**Affiliations:** a Organische Chemie, Ruhr-Universität Bochum, Universitätsstrasse 150, 44780 Bochum, Germany; b Analytische Chemie, Ruhr-Universität Bochum, Universitätsstrasse 150, 44780 Bochum, Germany

## Abstract

The crystal structure of the title compound, C_30_H_30_O_4_, comprises two crystallographically independent half-mol­ecules which are completed by crystallographic twofold symmetry. The dihedral angles between the naphthalene ring planes are 85.83 (3) and 83.69 (3)° for the two molecules. The atoms of the *tert*-butyl group of one mol­ecule are disordered over two sets of sites with occupancies of 0.60:0.40. The crystal packing is achieved *via* π–π stacking inter­actions between the naphthyl groups of adjacent mol­ecules, with a separation of 3.790 (1) Å between the centroids of the rings.

## Related literature

For the crystal structure of the parent (*R*)-2,2′-dihydr­oxy-1,1′-binaphthyl (BINOL), see: Mori *et al.* (1993 ▶). For the synthesis of the corresponding monopivalate of (*S*)-BINOL, see: Hocke & Uozumi (2002 ▶, 2003 ▶). For applications of BINOL-derived chiral ligands, see: Shibasaki & Matsunaga (2006 ▶).
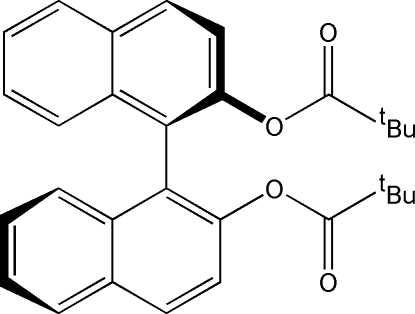


## Experimental

### 

#### Crystal data

C_30_H_30_O_4_
*M* *_r_* = 454.54
Orthorhombic, 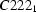

*a* = 9.6972 (4) Å
*b* = 25.8488 (13) Å
*c* = 19.9000 (9) Å
*V* = 4988.2 (4) Å^3^
*Z* = 8
Mo *K*α radiation
μ = 0.08 mm^−1^
*T* = 108 (2) K
0.37 × 0.21 × 0.18 mm

#### Data collection

Oxford Diffraction Sapphire2 CCD diffractometer
Absorption correction: multi-scan (ABSPACK in *CrysAlis RED*; Oxford Diffraction, 2008 ▶) *T*_min_ = 0.972, *T*_max_ = 0.984
33031 measured reflections
3175 independent reflections
2423 reflections with *I* > 2σ(*I*)
*R*_int_ = 0.043

#### Refinement

*R*[*F* ^2^ > 2σ(*F* ^2^)] = 0.033
*wR*(*F* ^2^) = 0.072
*S* = 0.89
3175 reflections
320 parameters
30 restraints
H-atom parameters constrained
Δρ_max_ = 0.18 e Å^−3^
Δρ_min_ = −0.16 e Å^−3^

### 

Data collection: *CrysAlis CCD* (Oxford Diffraction, 2008 ▶); cell refinement: *CrysAlis RED* (Oxford Diffraction, 2008 ▶); data reduction: *CrysAlis RED*; program(s) used to solve structure: *SHELXS97* (Sheldrick, 2008 ▶); program(s) used to refine structure: *SHELXL97* (Sheldrick, 2008 ▶); molecular graphics: *DIAMOND* (Brandenburg, 2008 ▶); software used to prepare material for publication: *SHELXL97*.

## Supplementary Material

Crystal structure: contains datablocks I, global. DOI: 10.1107/S1600536808044309/dn2418sup1.cif

Structure factors: contains datablocks I. DOI: 10.1107/S1600536808044309/dn2418Isup2.hkl

Additional supplementary materials:  crystallographic information; 3D view; checkCIF report

## Figures and Tables

**Fig. 1.**
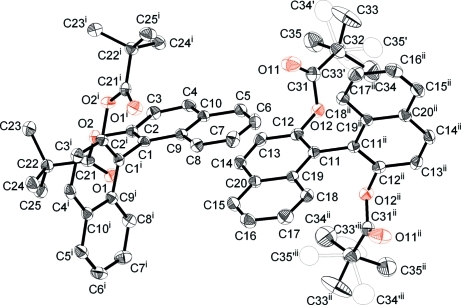
ORTEP diagram of the title compound. Displacement ellipsoids are drawn at the 50% probability level. H atoms are omitted for clarity. Symmetry codes: (i) -x + 2, y, -z + 1/2, (ii) x, -y + 1, -z + 1.

## supplementary crystallographic information

### Comment

For the (*S*)-form of 2,2'-dihydroxy-1,1'-binaphthyl (BINOL) was reported that during the reaction with equimolar amounts of pivaloyl chloride the formation of the corresponding 2,2'-pivalate is almost completely suppressed due to the steric hindrance rising from the bulky pivaloyl group (Hocke & Uozumi, 2002, 2003). However, we obtained the (*R*)-form of the 2,2'-dipivalate under forced conditions (see below) and determined its structure by X-ray crystallography.

An *ORTEP* diagram of the title compound is given in Fig. 1. The structure consists of two crystallographic independent molecules, which are located both on crystallographic twofold axes. The molecules belong to the point group *C*_2_ and exhibit axial chirality. Molecular geometry parameters are within expected ranges. The tertiary butyl groups of one molecule show rotational disorder over two sites with occupancies of 0.60:0.40. The dihedral angles between the mean planes of the naphthyl groups are 85.83 (3) and 83.69 (3)° for the non-disordered and the disordered molecule, respectively. In comparison, an angle of 78.35 (5)° has been reported for the parent (*R*)-BINOL (Mori *et al.*, 1993). The molecules interact *via*π–π stacking of the naphthyl groups of adjacent molecules with a separation of 3.790 (1) Å between the centroids of the rings C5–C10 and C15–C20. The mean interplanar distance is 3.59 Å and the offset is about 18.9°.

### Experimental

To a solution of (*R*)-BINOL (4.34 mmol, 1.26 g) and triethylamine (26.7 mmol, 3.7 ml) in acetonitrile (13 ml) was added pivaloyl chloride (8.94 mmol, 1.1 ml) dropwise at 0°C. Subsequent, the reaction mixture was stirred over night and allowed to warm up to room temperature. Diethyl ether was added and the mixture was washed with aqueous 1 N HCl, saturated aqueous NaHCO_3_ and brine three times, respectively. The organic phase was dried over MgSO_4_ and the solvent was removed under reduced pressure. The crude product was purified by column chromatography over silica gel using ethyl acetate–*n*-hexane (1:6) as eluent. Yield: 1.15 g (58%). Single crystals of the title compound suitable for X-ray diffraction were grown from ethyl acetate–*n*-hexane (1:6) by slow evaporation of the solvent.

[α]^25^_D_ + 88.94 (c 1.88, THF); ^1^H NMR (200 MHz, CDCl_3_) δ 7.30–7.37 (m, 4 H), 7.41–7.54 (m, 4 H), 7.96 (d, J = 8.17 Hz, 2 H), 8.01 (d, J = 8.93 Hz, 2 H); ^13^C NMR (200 MHz, CDCl_3_) δ 26.33, 38.61, 121.89, 123.62, 125.54, 126.03, 126.56, 127.82, 129.16, 131.44, 133.43, 146.94, 176.31; Anal. calcd for C_30_H_30_O_4_: C 79.27; H 6.65. Found: C 79.33, H 6.14; MS(FAB): *m*/*z* 455.3 [*M*+H]^+^.

### Refinement

The crystal structure was refined by full-matrix least-squares refinement on F^2^. Due to the absence of significant anomalous scattering effects, Friedel pairs have been merged. Anisotropic displacement parameters were introduced for all non-hydrogen atoms. Similar distance restraints were applied to the 1,2- and 1,3-distances of the disordered tertiary butyl group, respectively. The opposite C atoms of the disordered group were refined with equivalent anisotropic displacement parameters, respectively. H atoms were placed at geometrically calculated positions and refined with *U*_iso_ 1.2 times (1.5 for methyl groups) of their parent atoms and allowing to ride on them. The initial torsion angles of the methyl groups of the non-disordered tertiary butyl group were determined *via* a difference Fourier analysis. For the disordered tertiary butyl group those were calculated to be staggered.

### Crystal data

**Table:**

C_30_H_30_O_4_	*F*(000) = 1936
*M**_r_* = 454.54	*D*_x_ = 1.211 Mg m^−^^3^
Orthorhombic, *C*222_1_	Mo *K*α radiation, λ = 0.71073 Å
Hall symbol: C 2c 2	Cell parameters from 8276 reflections
*a* = 9.6972 (4) Å	θ = 2.6–30.3°
*b* = 25.8488 (13) Å	µ = 0.08 mm^−^^1^
*c* = 19.9000 (9) Å	*T* = 108 K
*V* = 4988.2 (4) Å^3^	Prism, colourless
*Z* = 8	0.37 × 0.21 × 0.18 mm

### Data collection

**Table:**

Oxford Diffraction Sapphire2 CCD diffractometer	3175 independent reflections
Radiation source: Enhance (Mo) X-ray Source	2423 reflections with *I* > 2σ(*I*)
graphite	*R*_int_ = 0.043
Detector resolution: 8.4171 pixels mm^-1^	θ_max_ = 27.5°, θ_min_ = 3.3°
ω scans	*h* = −12→12
Absorption correction: multi-scan (ABSPACK in *CrysAlis RED*; Oxford Diffraction, 2008)	*k* = −31→33
*T*_min_ = 0.972, *T*_max_ = 0.984	*l* = −25→25
33031 measured reflections	

### Refinement

**Table:**

Refinement on *F*^2^	Primary atom site location: structure-invariant direct methods
Least-squares matrix: full	Secondary atom site location: difference Fourier map
*R*[*F*^2^ > 2σ(*F*^2^)] = 0.033	Hydrogen site location: inferred from neighbouring sites
*wR*(*F*^2^) = 0.072	H-atom parameters constrained
*S* = 0.89	*w* = 1/[σ^2^(*F*_o_^2^) + (0.0436*P*)^2^] where *P* = (*F*_o_^2^ + 2*F*_c_^2^)/3
3175 reflections	(Δ/σ)_max_ < 0.001
320 parameters	Δρ_max_ = 0.18 e Å^−^^3^
30 restraints	Δρ_min_ = −0.16 e Å^−^^3^

### Special details

**Table:**

Geometry. All e.s.d.'s (except the e.s.d. in the dihedral angle between two l.s. planes) are estimated using the full covariance matrix. The cell e.s.d.'s are taken into account individually in the estimation of e.s.d.'s in distances, angles and torsion angles; correlations between e.s.d.'s in cell parameters are only used when they are defined by crystal symmetry. An approximate (isotropic) treatment of cell e.s.d.'s is used for estimating e.s.d.'s involving l.s. planes.
Refinement. Refinement of *F*^2^ against ALL reflections. The weighted *R*-factor *wR* and goodness of fit *S* are based on *F*^2^, conventional *R*-factors *R* are based on *F*, with *F* set to zero for negative *F*^2^. The threshold expression of *F*^2^ > σ(*F*^2^) is used only for calculating *R*-factors(gt) *etc*. and is not relevant to the choice of reflections for refinement. *R*-factors based on *F*^2^ are statistically about twice as large as those based on *F*, and *R*- factors based on ALL data will be even larger.

### Fractional atomic coordinates and isotropic or equivalent isotropic displacement parameters (Å^2^)

**Table:**

	*x*	*y*	*z*	*U*_iso_*/*U*_eq_	Occ. (<1)
O1	0.63715 (16)	0.30623 (6)	0.17450 (7)	0.0318 (4)	
O2	0.82275 (13)	0.26221 (5)	0.21109 (6)	0.0232 (3)	
C21	0.7200 (2)	0.27252 (8)	0.16612 (10)	0.0218 (4)	
C1	0.94407 (19)	0.32395 (7)	0.27619 (9)	0.0199 (4)	
C2	0.8298 (2)	0.29383 (7)	0.26837 (9)	0.0208 (4)	
C3	0.7240 (2)	0.29102 (8)	0.31619 (10)	0.0252 (5)	
H3	0.6466	0.2692	0.3090	0.030*	
C4	0.7338 (2)	0.31991 (8)	0.37300 (10)	0.0260 (5)	
H4	0.6630	0.3179	0.4059	0.031*	
C5	0.8550 (2)	0.38512 (8)	0.44130 (10)	0.0289 (5)	
H5	0.7839	0.3837	0.4741	0.035*	
C6	0.9627 (2)	0.41821 (9)	0.45025 (10)	0.0326 (5)	
H6	0.9656	0.4400	0.4887	0.039*	
C7	1.0696 (2)	0.42013 (9)	0.40251 (11)	0.0321 (5)	
H7	1.1449	0.4431	0.4091	0.039*	
C8	1.0663 (2)	0.38929 (8)	0.34679 (10)	0.0257 (5)	
H8	1.1398	0.3908	0.3153	0.031*	
C9	0.9546 (2)	0.35511 (8)	0.33540 (10)	0.0215 (4)	
C10	0.8474 (2)	0.35282 (8)	0.38388 (9)	0.0224 (4)	
C22	0.7294 (2)	0.23684 (8)	0.10603 (9)	0.0245 (5)	
C23	0.7295 (2)	0.18046 (8)	0.13026 (11)	0.0318 (5)	
H23A	0.8112	0.1743	0.1581	0.048*	
H23B	0.7313	0.1572	0.0913	0.048*	
H23C	0.6462	0.1739	0.1568	0.048*	
C24	0.8636 (2)	0.24911 (9)	0.06858 (11)	0.0334 (5)	
H24A	0.8635	0.2855	0.0548	0.050*	
H24B	0.8708	0.2270	0.0287	0.050*	
H24C	0.9424	0.2426	0.0983	0.050*	
C25	0.6065 (2)	0.24659 (9)	0.06012 (11)	0.0352 (5)	
H25A	0.5208	0.2400	0.0848	0.053*	
H25B	0.6116	0.2234	0.0212	0.053*	
H25C	0.6079	0.2826	0.0448	0.053*	
O11	0.49149 (19)	0.36700 (6)	0.55217 (8)	0.0481 (5)	
O12	0.35449 (14)	0.43270 (5)	0.52184 (7)	0.0263 (3)	
C11	0.53052 (19)	0.48267 (7)	0.46989 (9)	0.0210 (4)	
C12	0.4422 (2)	0.44153 (8)	0.46680 (10)	0.0224 (4)	
C13	0.4321 (2)	0.40866 (8)	0.41121 (10)	0.0263 (5)	
H13	0.3678	0.3809	0.4111	0.032*	
C14	0.5153 (2)	0.41686 (8)	0.35726 (10)	0.0270 (5)	
H14	0.5077	0.3950	0.3190	0.032*	
C15	0.7071 (2)	0.46426 (9)	0.30391 (10)	0.0324 (5)	
H15	0.7028	0.4419	0.2660	0.039*	
C16	0.8038 (2)	0.50233 (9)	0.30582 (11)	0.0351 (6)	
H16	0.8680	0.5058	0.2700	0.042*	
C17	0.8088 (2)	0.53657 (9)	0.36088 (11)	0.0344 (5)	
H17	0.8754	0.5635	0.3616	0.041*	
C18	0.7185 (2)	0.53139 (8)	0.41322 (11)	0.0267 (5)	
H18	0.7222	0.5551	0.4496	0.032*	
C19	0.6200 (2)	0.49123 (7)	0.41380 (9)	0.0215 (4)	
C20	0.6130 (2)	0.45738 (8)	0.35732 (9)	0.0244 (5)	
C31	0.3931 (2)	0.39330 (9)	0.56367 (10)	0.0292 (5)	
C32	0.2992 (3)	0.38865 (9)	0.62358 (12)	0.0388 (6)	
C33	0.3695 (5)	0.3673 (3)	0.6811 (2)	0.0810 (18)	0.601 (3)
H33A	0.3049	0.3646	0.7188	0.122*	0.601 (3)
H33B	0.4464	0.3899	0.6936	0.122*	0.601 (3)
H33C	0.4048	0.3328	0.6699	0.122*	0.601 (3)
C34	0.2190 (6)	0.4386 (2)	0.6369 (3)	0.0647 (13)	0.601 (3)
H34A	0.1620	0.4469	0.5977	0.097*	0.601 (3)
H34B	0.2840	0.4670	0.6452	0.097*	0.601 (3)
H34C	0.1598	0.4340	0.6764	0.097*	0.601 (3)
C35	0.1845 (5)	0.34794 (19)	0.5970 (2)	0.0549 (10)	0.601 (3)
H35A	0.1364	0.3626	0.5581	0.082*	0.601 (3)
H35B	0.1180	0.3410	0.6329	0.082*	0.601 (3)
H35C	0.2299	0.3156	0.5838	0.082*	0.601 (3)
C33'	0.1614 (7)	0.4053 (5)	0.6191 (4)	0.0810 (18)	0.399 (3)
H33D	0.1151	0.3996	0.6622	0.122*	0.399 (3)
H33E	0.1139	0.3858	0.5838	0.122*	0.399 (3)
H33F	0.1593	0.4423	0.6081	0.122*	0.399 (3)
C34'	0.3208 (10)	0.3352 (3)	0.6572 (4)	0.0647 (13)	0.399 (3)
H34D	0.4194	0.3270	0.6582	0.097*	0.399 (3)
H34E	0.2718	0.3086	0.6314	0.097*	0.399 (3)
H34F	0.2848	0.3361	0.7032	0.097*	0.399 (3)
C35'	0.3811 (7)	0.4262 (3)	0.6770 (3)	0.0549 (10)	0.399 (3)
H35D	0.4773	0.4150	0.6807	0.082*	0.399 (3)
H35E	0.3368	0.4239	0.7212	0.082*	0.399 (3)
H35F	0.3779	0.4620	0.6611	0.082*	0.399 (3)

### Atomic displacement parameters (Å^2^)

**Table:**

	*U*^11^	*U*^22^	*U*^33^	*U*^12^	*U*^13^	*U*^23^
O1	0.0349 (8)	0.0306 (8)	0.0298 (8)	0.0125 (7)	−0.0038 (7)	−0.0014 (6)
O2	0.0208 (7)	0.0249 (8)	0.0238 (7)	0.0003 (6)	−0.0012 (6)	−0.0050 (6)
C21	0.0191 (10)	0.0226 (11)	0.0238 (10)	−0.0024 (9)	0.0019 (8)	0.0038 (9)
C1	0.0190 (10)	0.0223 (11)	0.0185 (9)	0.0035 (8)	0.0001 (8)	0.0034 (8)
C2	0.0204 (10)	0.0224 (10)	0.0197 (10)	0.0020 (8)	−0.0021 (8)	−0.0008 (8)
C3	0.0206 (10)	0.0265 (12)	0.0284 (11)	−0.0013 (9)	0.0030 (9)	0.0020 (9)
C4	0.0259 (11)	0.0295 (12)	0.0228 (10)	0.0040 (10)	0.0086 (9)	0.0058 (9)
C5	0.0333 (12)	0.0321 (12)	0.0213 (10)	0.0091 (10)	0.0022 (10)	0.0004 (9)
C6	0.0387 (13)	0.0331 (13)	0.0260 (11)	0.0116 (11)	−0.0052 (10)	−0.0085 (10)
C7	0.0274 (11)	0.0322 (13)	0.0368 (12)	0.0021 (10)	−0.0070 (10)	−0.0072 (10)
C8	0.0223 (11)	0.0290 (12)	0.0258 (11)	0.0017 (9)	0.0002 (9)	−0.0009 (9)
C9	0.0218 (10)	0.0225 (11)	0.0201 (10)	0.0040 (8)	−0.0015 (8)	0.0012 (8)
C10	0.0253 (11)	0.0223 (11)	0.0195 (10)	0.0055 (9)	0.0000 (8)	0.0025 (8)
C22	0.0252 (11)	0.0251 (12)	0.0232 (10)	0.0012 (9)	0.0010 (9)	−0.0014 (8)
C23	0.0377 (13)	0.0269 (12)	0.0307 (11)	−0.0019 (10)	0.0000 (10)	−0.0033 (9)
C24	0.0345 (12)	0.0383 (13)	0.0275 (11)	0.0004 (11)	0.0090 (10)	−0.0041 (9)
C25	0.0387 (13)	0.0371 (14)	0.0297 (12)	0.0043 (11)	−0.0083 (10)	−0.0058 (10)
O11	0.0449 (10)	0.0526 (11)	0.0469 (10)	0.0205 (9)	0.0125 (9)	0.0172 (9)
O12	0.0248 (8)	0.0240 (8)	0.0302 (7)	0.0000 (6)	0.0037 (6)	0.0000 (6)
C11	0.0211 (10)	0.0209 (11)	0.0211 (10)	0.0035 (8)	−0.0023 (8)	−0.0003 (8)
C12	0.0194 (10)	0.0217 (11)	0.0260 (11)	0.0037 (8)	0.0002 (9)	0.0017 (8)
C13	0.0256 (11)	0.0216 (11)	0.0319 (11)	−0.0002 (9)	−0.0062 (9)	−0.0026 (9)
C14	0.0311 (12)	0.0272 (12)	0.0227 (10)	0.0051 (10)	−0.0078 (9)	−0.0051 (9)
C15	0.0398 (13)	0.0362 (13)	0.0214 (11)	0.0079 (11)	0.0009 (10)	−0.0022 (10)
C16	0.0327 (12)	0.0445 (14)	0.0282 (12)	0.0059 (11)	0.0101 (10)	0.0055 (10)
C17	0.0314 (12)	0.0330 (13)	0.0388 (13)	−0.0035 (10)	0.0059 (11)	0.0024 (11)
C18	0.0293 (11)	0.0238 (11)	0.0271 (11)	−0.0007 (10)	0.0013 (9)	−0.0010 (9)
C19	0.0227 (10)	0.0218 (11)	0.0201 (10)	0.0044 (9)	−0.0015 (9)	0.0008 (8)
C20	0.0277 (11)	0.0248 (11)	0.0208 (10)	0.0062 (9)	−0.0030 (9)	−0.0001 (9)
C31	0.0285 (12)	0.0292 (12)	0.0298 (11)	−0.0034 (10)	−0.0030 (10)	−0.0011 (10)
C32	0.0392 (13)	0.0397 (14)	0.0377 (12)	−0.0006 (12)	0.0118 (11)	0.0038 (11)
C33	0.045 (2)	0.161 (6)	0.037 (2)	0.008 (3)	0.006 (2)	0.036 (3)
C34	0.072 (3)	0.063 (3)	0.060 (3)	0.002 (2)	0.034 (3)	−0.003 (2)
C35	0.055 (2)	0.062 (3)	0.048 (2)	−0.018 (2)	0.012 (2)	−0.0006 (19)
C33'	0.045 (2)	0.161 (6)	0.037 (2)	0.008 (3)	0.006 (2)	0.036 (3)
C34'	0.072 (3)	0.063 (3)	0.060 (3)	0.002 (2)	0.034 (3)	−0.003 (2)
C35'	0.055 (2)	0.062 (3)	0.048 (2)	−0.018 (2)	0.012 (2)	−0.0006 (19)

### Geometric parameters (Å, °)

**Table:**

O1—C21	1.197 (2)	C12—C13	1.398 (3)
O2—C21	1.365 (2)	C13—C14	1.360 (3)
O2—C2	1.404 (2)	C13—H13	0.9500
C21—C22	1.513 (3)	C14—C20	1.412 (3)
C1—C2	1.363 (3)	C14—H14	0.9500
C1—C9	1.431 (3)	C15—C16	1.360 (3)
C1—C1^i^	1.504 (4)	C15—C20	1.412 (3)
C2—C3	1.401 (3)	C15—H15	0.9500
C3—C4	1.358 (3)	C16—C17	1.409 (3)
C3—H3	0.9500	C16—H16	0.9500
C4—C10	1.408 (3)	C17—C18	1.367 (3)
C4—H4	0.9500	C17—H17	0.9500
C5—C6	1.361 (3)	C18—C19	1.411 (3)
C5—C10	1.417 (3)	C18—H18	0.9500
C5—H5	0.9500	C19—C20	1.426 (3)
C6—C7	1.407 (3)	C31—C32	1.505 (3)
C6—H6	0.9500	C32—C33'	1.407 (7)
C7—C8	1.366 (3)	C32—C33	1.441 (5)
C7—H7	0.9500	C32—C34	1.531 (5)
C8—C9	1.417 (3)	C32—C34'	1.549 (7)
C8—H8	0.9500	C32—C35	1.620 (5)
C9—C10	1.419 (3)	C32—C35'	1.644 (6)
C22—C25	1.522 (3)	C33—H33A	0.9800
C22—C24	1.533 (3)	C33—H33B	0.9800
C22—C23	1.535 (3)	C33—H33C	0.9800
C23—H23A	0.9800	C34—H34A	0.9800
C23—H23B	0.9800	C34—H34B	0.9800
C23—H23C	0.9800	C34—H34C	0.9800
C24—H24A	0.9800	C35—H35A	0.9800
C24—H24B	0.9800	C35—H35B	0.9800
C24—H24C	0.9800	C35—H35C	0.9800
C25—H25A	0.9800	C33'—H33D	0.9800
C25—H25B	0.9800	C33'—H33E	0.9800
C25—H25C	0.9800	C33'—H33F	0.9800
O11—C31	1.194 (3)	C34'—H34D	0.9800
O12—C31	1.368 (2)	C34'—H34E	0.9800
O12—C12	1.405 (2)	C34'—H34F	0.9800
C11—C12	1.367 (3)	C35'—H35D	0.9800
C11—C19	1.431 (3)	C35'—H35E	0.9800
C11—C11^ii^	1.496 (4)	C35'—H35F	0.9800
			
C21—O2—C2	117.00 (15)	C15—C16—H16	120.0
O1—C21—O2	122.72 (18)	C17—C16—H16	120.0
O1—C21—C22	126.45 (18)	C18—C17—C16	120.6 (2)
O2—C21—C22	110.82 (16)	C18—C17—H17	119.7
C2—C1—C9	118.25 (17)	C16—C17—H17	119.7
C2—C1—C1^i^	120.46 (17)	C17—C18—C19	120.8 (2)
C9—C1—C1^i^	121.29 (17)	C17—C18—H18	119.6
C1—C2—C3	123.17 (18)	C19—C18—H18	119.6
C1—C2—O2	117.69 (17)	C18—C19—C20	118.49 (18)
C3—C2—O2	119.05 (17)	C18—C19—C11	122.06 (17)
C4—C3—C2	119.06 (19)	C20—C19—C11	119.42 (18)
C4—C3—H3	120.5	C14—C20—C15	121.75 (18)
C2—C3—H3	120.5	C14—C20—C19	119.18 (18)
C3—C4—C10	120.98 (19)	C15—C20—C19	119.03 (19)
C3—C4—H4	119.5	O11—C31—O12	121.7 (2)
C10—C4—H4	119.5	O11—C31—C32	126.1 (2)
C6—C5—C10	121.0 (2)	O12—C31—C32	112.11 (19)
C6—C5—H5	119.5	C33'—C32—C33	128.1 (4)
C10—C5—H5	119.5	C33'—C32—C31	120.0 (4)
C5—C6—C7	119.94 (19)	C33—C32—C31	111.9 (3)
C5—C6—H6	120.0	C33'—C32—C34	43.1 (5)
C7—C6—H6	120.0	C33—C32—C34	115.2 (4)
C8—C7—C6	120.7 (2)	C31—C32—C34	112.2 (3)
C8—C7—H7	119.7	C33'—C32—C34'	115.4 (6)
C6—C7—H7	119.7	C33—C32—C34'	41.6 (4)
C7—C8—C9	120.79 (19)	C31—C32—C34'	109.4 (3)
C7—C8—H8	119.6	C34—C32—C34'	138.3 (4)
C9—C8—H8	119.6	C33'—C32—C35	61.7 (5)
C8—C9—C10	118.51 (17)	C33—C32—C35	109.5 (4)
C8—C9—C1	122.49 (17)	C31—C32—C35	102.0 (2)
C10—C9—C1	118.97 (17)	C34—C32—C35	104.8 (3)
C4—C10—C5	121.36 (19)	C34'—C32—C35	69.8 (4)
C4—C10—C9	119.55 (17)	C33'—C32—C35'	108.6 (6)
C5—C10—C9	119.05 (19)	C33—C32—C35'	59.0 (4)
C21—C22—C25	109.06 (16)	C31—C32—C35'	100.0 (3)
C21—C22—C24	107.99 (16)	C34—C32—C35'	68.6 (4)
C25—C22—C24	109.79 (16)	C34'—C32—C35'	100.5 (5)
C21—C22—C23	109.30 (16)	C35—C32—C35'	157.8 (3)
C25—C22—C23	110.28 (18)	C32—C33—H33A	109.5
C24—C22—C23	110.37 (18)	C32—C33—H33B	109.5
C22—C23—H23A	109.5	H33A—C33—H33B	109.5
C22—C23—H23B	109.5	C32—C33—H33C	109.5
H23A—C23—H23B	109.5	H33A—C33—H33C	109.5
C22—C23—H23C	109.5	H33B—C33—H33C	109.5
H23A—C23—H23C	109.5	C32—C34—H34A	109.5
H23B—C23—H23C	109.5	C32—C34—H34B	109.5
C22—C24—H24A	109.5	H34A—C34—H34B	109.5
C22—C24—H24B	109.5	C32—C34—H34C	109.5
H24A—C24—H24B	109.5	H34A—C34—H34C	109.5
C22—C24—H24C	109.5	H34B—C34—H34C	109.5
H24A—C24—H24C	109.5	C32—C35—H35A	109.5
H24B—C24—H24C	109.5	C32—C35—H35B	109.5
C22—C25—H25A	109.5	H35A—C35—H35B	109.5
C22—C25—H25B	109.5	C32—C35—H35C	109.5
H25A—C25—H25B	109.5	H35A—C35—H35C	109.5
C22—C25—H25C	109.5	H35B—C35—H35C	109.5
H25A—C25—H25C	109.5	C32—C33'—H33D	109.5
H25B—C25—H25C	109.5	C32—C33'—H33E	109.5
C31—O12—C12	115.44 (15)	H33D—C33'—H33E	109.5
C12—C11—C19	117.72 (17)	C32—C33'—H33F	109.5
C12—C11—C11^ii^	120.13 (17)	H33D—C33'—H33F	109.5
C19—C11—C11^ii^	122.14 (17)	H33E—C33'—H33F	109.5
C11—C12—C13	123.51 (19)	C32—C34'—H34D	109.5
C11—C12—O12	118.07 (17)	C32—C34'—H34E	109.5
C13—C12—O12	118.40 (18)	H34D—C34'—H34E	109.5
C14—C13—C12	119.23 (19)	C32—C34'—H34F	109.5
C14—C13—H13	120.4	H34D—C34'—H34F	109.5
C12—C13—H13	120.4	H34E—C34'—H34F	109.5
C13—C14—C20	120.87 (18)	C32—C35'—H35D	109.5
C13—C14—H14	119.6	C32—C35'—H35E	109.5
C20—C14—H14	119.6	H35D—C35'—H35E	109.5
C16—C15—C20	121.1 (2)	C32—C35'—H35F	109.5
C16—C15—H15	119.5	H35D—C35'—H35F	109.5
C20—C15—H15	119.5	H35E—C35'—H35F	109.5
C15—C16—C17	120.0 (2)		

Symmetry codes: (i) −*x*+2, *y*, −*z*+1/2; (ii) *x*, −*y*+1, −*z*+1.
